# Actions, indicators, and outputs in urban biodiversity plans: A multinational analysis of city practice

**DOI:** 10.1371/journal.pone.0235773

**Published:** 2020-07-08

**Authors:** Jennifer Rae Pierce, Melissa A. Barton, Mika Mei Jia Tan, Ginevra Oertel, Michael D. Halder, Pablo Arturo Lopez-Guijosa, Rohan Nuttall

**Affiliations:** 1 Urban Biodiversity Hub, Vancouver, BC, Canada; 2 School of Community and Regional Planning, University of British Columbia, Vancouver, BC, Canada; 3 ASEAN Youth Biodiversity Programme, ASEAN Biodiversity Centre, Los Baños, Philippines; 4 Department of Environmental Sciences and Policy, Central European University, Budapest, Hungary; 5 Department of Computing Science, University of Alberta, Edmonton, AB, Canada; Leiden University, NETHERLANDS

## Abstract

Urban biodiversity offers important benefits to residents and may be crucial to reaching global biodiversity conservation targets, but little research has been conducted on how cities actually plan for biodiversity. In this study, we conducted a mixed methods content analysis of biodiversity plans by 39 cities around the world to determine whether they measured their actions, how they did so (via quantitative indicators and qualitative outputs), and what topics these actions and measures covered. We based our analytical framework on the Singapore Index on Cities’ Biodiversity (also known as the City Biodiversity Index), a widely applied 23-indicator index that helps cities track their progress in biodiversity planning. The Singapore Index groups its indicators into the following three core components: native biodiversity, ecosystem services, and governance and management. For actions and measures not classifiable by the Singapore Index, we inductively derived additional categories. Across all plans, we identified 2,231 actions, 346 indicators, and 444 outputs. We found that all of the plans included actions, while 82% included measures (67% included indicators and 72% included outputs). Only 29% of actions were associated with a measure. Overall, the plans covered all of the categories in the Singapore Index, particularly within the core components of native biodiversity and governance and management, though some plans had a narrower focus. The 20 additional urban biodiversity topics that were not covered by the Singapore Index framework included socioeconomic considerations, data collection, genetic diversity, urban agriculture and forestry, green infrastructure, human-wildlife conflicts, indigenous concerns, and citizen science. Indicators were the most common measures for native biodiversity and ecosystem service topics, while outputs were the most common measures for governance and management. Our results may inform the revision and development of urban biodiversity indicators in the post-2020 framework and of other initiatives that guide cities in contributing to local and global biodiversity goals.

## Introduction

Today, more than half of the world’s population lives in cities, and the United Nations projects this percentage to reach 68% by 2050 [1]. Biodiversity contributes significantly to human wellbeing [2], and its loss adversely impacts urban dwellers as well as others [3]. Conversely, urbanites have both local and long-ranging impact on biodiversity, as indicated by their large ecological footprints [4, 5] and the telecoupled impacts of cities across long distances [6]. However, recent research on nature-based solutions [7–9] has recognized opportunities to both provide a better quality of life to urban residents and reduce the ecological footprint of cities through biodiversity planning practices such as integrating nature into the urban fabric.

The year 2020 is a critical year for global biodiversity governance. As the United Nations Decade on Biodiversity 2011–2020 and the Strategic Plan for Biodiversity 2011–2020 come to an end in 2020, the world’s governments are discussing the successor to the Strategic Plan, which is known as the post-2020 global biodiversity framework [10, 11]. As stakeholders consult on goals, targets, and indicators for this post-2020 global biodiversity framework, they have called for specific goals related to urban conservation efforts, including urban growth targets or urban biodiversity metrics that measure progress against global targets [3]. In 2008, the Parties to the Convention on Biological Diversity (CBD) agreed on Decision IX/28, which called for parties to support biodiversity efforts by cities [12]. Despite adopting a mandate for national biodiversity strategies, the CBD has yet to adopt a similar mandate for local biodiversity plans. Some nations, such as Japan and the United Kingdom, have mandated them at the city level, and even without a mandate, some cities began developing biodiversity plans or strategies at least as early as the 1990s [13]. Yet, thus far, relatively little research has been conducted on how governments track these efforts or on how such metrics on urban biodiversity might be incorporated into planning at the city level.

Planning for biodiversity can be in the form of any combination of (i) specific biodiversity strategies, habitat plans, and/or ecological health plans and/or (ii) integration of biodiversity into other city plans (e.g., sustainability, stormwater, and green area plans). A recent report summarizing the state of urban biodiversity identified 108 local governments that had released biodiversity plans or strategies, exclusive of biodiversity initiatives integrated into comprehensive plans [3]. More than one-third (45/108) of these urban biodiversity plans were produced by cities in Europe; cities in North America and Asia produced 23 each [3]. Japan and the United Kingdom were responsible for many of these plans [13]. Other studies of urban biodiversity planning have examined planning at the local level (e.g., Ahmed and Puppim de Oliveira [14]). However, producing a plan or strategy specific to biodiversity (or related topics) remains an uncommon practice for most cities (e.g., Miller et al. [15]). A recent survey of experts in 25 countries found that one of the barriers is a lack of technical knowledge on the topic [16]. This suggests that strengthening frameworks and tools for these areas will be an important part of improving urban biodiversity planning. Sandström et al. [17] identified similar barriers to integration of biodiversity into comprehensive planning in Swedish cities.

Currently, there are more than 22 frameworks specific to urban biodiversity that have been applied internationally, but no widespread standards or reporting mechanisms have taken hold at the global level [3]. Many of these frameworks are still being actively developed or used, sometimes in competition with one another. This variation in approaches is reflected in biodiversity plans, which also differ widely in their contents [18] and even in how they define biodiversity itself [13]. These plans have also been critiqued for their lack of standards and accountability measures [19], such as targets [18], which may be linked to the general lack of a standard approach for biodiversity conservation [20]. Given the lack of standard frameworks, the variety of biodiversity planning approaches, and the lack of standardized measurements for biodiversity plans, developing these plans can be challenging for city governments.

Measurable indicators with accompanying targets and/or verifiable outputs are important for determining whether planning goals have been achieved. However, understanding of how these measures are actually included in urban biodiversity plans is needed. Hence, we focused our study on better understanding how cities state their intentions to measure their progress on biodiversity actions through qualitative and quantitative measures defined as outputs and indicators, respectively. We selected the Singapore Index on Cities’ Biodiversity (SI), also known as the City Biodiversity Index (CBI) [21], as our primary analytical framework because of its global application, coverage of a wide range of urban biodiversity topics, and planned update in the near future. Using the SI as a framework, we investigated how cities address biodiversity conservation through explicitly stated actions and measures in their plans. Thus, in this study, we seek to address the following questions:

- In their biodiversity plans, are cities explicitly stating their intended actions and linking these statements to accountability measures?
- Are the SI indicator categories a representative framework for actions and measures in urban biodiversity plans?
- What topics are covered in urban biodiversity plans and how are they measured?

Through this analysis of current practice, we further elucidate the state of urban biodiversity planning in cities by describing the approaches cities are taking to promote and measure urban biodiversity. We also offer a guide for refinement and development of future urban biodiversity indicators and indices by identifying which topics are most and least frequently measured.

## Materials and methods

We analyzed biodiversity plans from a sample of geographically distributed cities using a mixed-methods content analysis of plan elements coded by a framework of Singapore Index indicators. If no Singapore Index indicator was appropriate for any particular plan element, we inductively derived additional coding categories (topic areas) from the plans themselves. The unit of analysis in this study was the plan, with descriptive statistics and analysis focused primarily at this level.

Content analysis uses systematic methods based upon a coding protocol that results in dependable and replicable quantitative attribute data that describe the media of interest [22]. We used a comparative approach that has been recognized previously as appropriate for evaluating planning and policy [23, 24]. Content analysis is a recognized method for comparative plan studies, for which there is an existing methodological framework within the planning literature that we adapted for our study [24]. We adapted the categories of the SI for our framework but did not incorporate its scoring and specific calculation methods, since we were neither scoring the cities’ plans nor assessing their quality. We used manual qualitative content analysis [22] to better understand and verify patterns observed in the quantitative content analysis. This involved reading the filtered results of the categorized plan elements to ensure consistent application of the coding protocol and to revise the inductively derived coding categories as necessary for clarity and consistency.

### Sample selection

Our study population consisted of cities with dedicated biodiversity plans. We identified these cities using the Urban Biodiversity Hub (UBHub) database (accessed 7 October 2019; http://ubhub.org/map), which is global in scope (1,386 locations) and is currently the most extensive publicly available database containing urban biodiversity plans (208 plans). The UBHub database has its roots in research that was conducted in 2011 for a master’s thesis by Pierce comparing biodiversity plans around the world [13]. Additional records have since been added by Pierce, with further contributions since 2016 by the UBHub team. Entries to the database were identified by one of the following strategies: (i) systematic internet searches by keyword (such as “biodiversity plan” or “green infrastructure plan” in several languages), (ii) particular searches within the websites of prominent city governments and biodiversity initiatives, (iii) literature searches, and (iv) word of mouth suggestions and user submissions. For our study, we defined “biodiversity plans” to include open space plans, green plans, habitat plans, and ecological health plans, but we did not include climate change action plans (these have been studied in depth elsewhere, e.g., by Reckien [25]) or comprehensive plans.

From the UBHub database, we identified 111 cities (including one city-state, Singapore, and one special administrative region, Hong Kong) that have created or adopted biodiversity plans, here referred to as “urban biodiversity plans.” From this population of cities, we selected biodiversity plans according to city population from largest to smallest, capping the number of plans in a given country at three to increase diversity. This strategy resulted in a sample of 39 cities in 24 countries, with city populations ranging from 375,000 to 24 million (Table 1; see S1 Table in the supporting information for plan titles and further metadata). We aimed to include comparable numbers of cities from each World Bank region (https://data.worldbank.org/country) in the sample, but for some regions we found few or no biodiversity plans (e.g., South Asia). Furthermore, we did not include plans from metro areas or districts (e.g., Washington, DC) or plans that were not officially incorporated into city documents (i.e., New York City’s Nature Goals).

**Table 1:** Regional distribution of the 39 cities included in this study.

World Bank region^a^	No. of cities	City	Country
**East Asia and Pacific**	10	Melbourne	Australia
		Sydney	Australia
		Hong Kong^c^	China
		Shanghai	China
		Singapore^c^	Singapore
		Nagoya	Japan
		Sapporo	Japan
		Yokohama	Japan
		Auckland	New Zealand
		Christchurch	New Zealand
**Europe and Central Asia**	15	Copenhagen	Denmark
		Birmingham	England, UK
		Leeds	England, UK
		London	England, UK
		Paris	France
		Berlin	Germany
		Hamburg	Germany
		Dublin	Ireland
		Amsterdam	Netherlands
		Oslo	Norway
		Lisbon	Portugal
		Edinburgh	Scotland, UK
		Glasgow	Scotland, UK
		Barcelona	Spain
		Zurich	Switzerland
**Latin America and the Caribbean**	4	São Paulo	Brazil
		Bogota	Colombia
		Medellin	Colombia
		Mexico City	Mexico
**Middle East and North Africa**	1	Jerusalem	Israel
**North America**	5	Chicago	USA
		San Diego	USA
		Calgary	Canada
		Montreal	Canada
		Toronto	Canada
**South Asia**^**b**^	0	N/A	N/A
**Sub-Saharan Africa**	4	Cape Town	South Africa
		eThekwini (Durban)	South Africa
		Johannesburg	South Africa
		Lilongwe	Malawi

^a^Note that the World Bank region of “North America” differs from the geographic region of the same name and includes only Bermuda, Canada, and the United States. Several other countries in this geographic region are included in the “Latin America” World Bank region.

^b^No plans from South Asia were available for analysis.

^c^Note that Hong Kong is a semi-autonomous Special Administrative Region of China and Singapore is a city-state.

### Analytical framework

We chose the Singapore Index as our analytical framework because it is one of the first urban biodiversity indicator systems developed, it is supported by the Convention on Biological Diversity, and it remains one of the most widely applied indicator systems for urban biodiversity worldwide. The SI provides a standardized framework for cities to make an initial baseline measurement of their biodiversity status and efforts via a quantitative index, and it encourages cities to use their results to identify opportunities for improvement and policy priorities [21].

The SI comprises 23 unique indicators covering a variety of topics, which are in turn categorized under the three core components of native biodiversity (NB), ecosystem services (ES), and governance and management (GM). The experiences of specific cities with applying the Singapore Index have been documented elsewhere [26–29]. In our study, we instead used the SI as a categorization framework to understand how urban biodiversity plans state and measure their goals, whether or not the cities in question have explicitly adopted and applied the SI itself. We therefore used a simplified version of the SI framework for coding (see S2 Table in the supporting material), as cities may not use the same detailed calculations and definitions as the formal SI indicators. Note that unless specifically described as an “SI indicator,” in this study, “indicators” refer to indicators found in the urban biodiversity plans.

### Coding protocol and analysis

After selecting the cities via the UBHub database, we searched online for the most recent plan that addresses biodiversity issues for each city. In some cases, biodiversity elements were incorporated into a larger sustainability or open space plan at a later date. In these cases, we analyzed the most recent document, but only those sections pertaining to biodiversity. Plans that were not available in English were reviewed by coders who had knowledge of the original language. Translations were supported by automated translation software (Google Translate) and by consultation with native speakers as necessary.

In each plan, we started by identifying intended actions where they had been clearly set apart, such as in a table or a bulleted list. Sometimes this required splitting a single statement with two embedded actions into two separately coded actions. Then we examined the plan for measures (indicators or outputs), which might be listed in a separate section or table but often were found together with or even integrated into the same sentence as a corresponding action. Since the terms “action,” “indicator,” and “output” are not standardized across (and sometimes even within) plans, we adopted definitions for each term as part of our coding protocol (Table 2). We extracted and reclassified elements of the plans as necessary for coding consistency.

**Table 2:** Definitions of plan elements.

Term	Definition	Example
**Action**	A specific action the city plans to take (or is taking), generally expressed with a verb.	“Carry out riparian water vole surveys.”
**Indicator**	A quantifiable measure based on verifiable data that is intended to convey information about the city’s progress in reaching a stated objective.^a^	“Number of research projects conducted.”
**Output**	A nonquantifiable measure of success or completion towards an objective that can be determined objectively, such as a deliverable or an event.	“Publish an agricultural biodiversity guide.”

^a^Modified from the Biodiversity Indicators Partnership definition (https://www.bipindicators.net/national-indicator-development), which defines an indicator as a “measure based on verifiable data that conveys information about more than just itself” and notes that the interpretation of indicators is purpose dependent.

These three types of plan elements were identified according to the definitions given in Table 3, regardless of the terms used in the plans themselves, and extracted. Each element was then coded manually in a spreadsheet according to the thematic matrix format [30]. Our coding framework was based on the structure of the Singapore Index. We began by defining extracted elements according to our standard definitions, then assigned each extract to one of the SI indicators, or, in some cases, with additional granularity. For example, SI indicators 7 and 8 each allow cities to choose an additional taxonomic category beyond the plants, birds, and butterflies covered by indicators 3 to 6, respectively. We separately recorded all taxonomic or ecological groups (e.g., fungi, mammals, or marine taxa) referenced in the plans, as well as references to species counts with no specified taxonomic group. For overall analysis, we grouped plan elements classified by SI indicators 3 to 8 and also included with them elements referring to general species counts (that did not specify a taxonomic group), since these had the same intent: that of estimating the presence of local species. Therefore, with the species-based SI indicators (3 to 8) treated as a group, we have a total of 18 categories rather than the original 23 indicators.

**Table 3:** Percentages of actions, indicators, and outputs in plans, along with percentages of actions with linked measures, classified by Singapore Index indicator.

SI indicator	Average % across plans of all			Overall proportion (%) of actions to	
	Actions (*n* = 39)	Indicators (*n* = 26)	Outputs (*n* = 28)	Indicators	Outputs
**Native biodiversity**					
**1. Natural areas**	6.4	20.1	4.4	32	10
**2. Connectivity**	7.3	3.3	4.1	3	45
**3–8. Native species counts**	6.2	9.5	3.6	24	10
**9. Protected natural areas**	3.8	5.0	1.2	22	22
**10. Invasive species**	3.2	5.5	4.8	21	18
**Ecosystem services**					
**11. Regulation of water quantity**	0.7	1.6	0.4	55	9
**12. Carbon storage and cooling effect of vegetation**	2.3	3.8	1.7	40	19
**13. Natural park areas**	3.4	1.9	1.3	13	13
**14. Visits to natural areas**	0.7	1.2	0.3	28	6
**Governance and management**					
**15. Budget allocation for biodiversity**	1.4	1.0	0.9	26	21
**16. Biodiversity projects**	1.2	5.0	4.7	22	35
**17. LBSAP**^**a**^	1.8	0.1	4.4	3	43
**18. Biodiversity-related functions**	0.4	0.7	2.0	38	54
**19. Interagency cooperation**	5.8	3.5	7.0	18	29
**20. Public participation**	2.0	1.2	4.4	12	26
**21. Partnerships**	5.3	2.5	4.5	10	20
**22. School curricula**	1.3	0.4	0.3	6	16
**23. Outreach**	7.2	4.7	5.6	13	11

^a^LBSAP, Local Biodiversity Strategy and Action Plan. This indicator refers to the creation of a biodiversity plan.

Each of the actions and measures were coded according to the perceived intent of the text (as determined by explicit objectives or similar statements in the plan), rather than by the method of measurement, which may rely on proxy indicators that could be used for multiple intents (e.g., area of green space as a proxy for carbon capture or as a measurement of permeable surface). In order to be classified by an SI indicator, the plan element had to explicitly state an intention of matching scope to that of the indicator or be measuring the same phenomenon as the indicator. It did not have to measure the intended phenomenon using the same mechanism as the SI. When an action or measure could be coded by more than one SI indicator, only the most relevant SI indicator was assigned to each to maintain simplicity for this analysis. According to recommended practices, we pretested the protocol on a sample of 4 plans and adjusted it before coding a larger set [24, 31]. These four plans constitute 10% of the 39 cities and are thus within the recommended range for pretesting of 10 to 20% of the sample [30].

The plan elements were first coded deductively, using the SI framework. If an element did not align with the SI framework, it was marked as not applicable (n/a) and subsequently categorized inductively according to themes emerging from the text. We maintained a living coding protocol spreadsheet shared by all coders to track inductive themes throughout the coding phase. To increase intercoder reliability among our team of six coders, each coder underwent training specific to the project methodology and coding protocol, and we maintained a shared journal entry for each plan describing the approach taken, following recommended practices [24, 31]. The lead author manually reviewed each coded element once the team members had independently coded the plans. The final coded data are available in S1 Dataset in the supporting information.

For analysis, we explored the data at the plan level according to the SI core components and indicators through visuals, pivot tables, and descriptive statistics. We then explored the relative frequency of the occurrence of the SI indicator topics in the plans. In order to determine the relative ratio of elements within each plan by SI indicator, we calculated the percentage for each element within each plan for each SI indicator, then took an average of this number across all of the plans. This was repeated for each indicator and type of plan element. Lastly, we filtered the elements by their coding and manually conducted more in-depth qualitative interpretation to reveal patterns which are described in our observations.

### Limitations

Although we attempted to obtain a globally representative sample of plans, we cannot claim comprehensive or unbiased coverage. In spite of using the most extensive publicly available database of plans and including plans in languages other than English, cities in areas of the world where planning documents are not available in English may have been undersampled.

Two-thirds of the selected plans (26 of 39) were available in English, at least in summary form. Actions and indicators in the remaining plans were translated into English for analysis. It is likely that some nuances of meaning were lost in the translation process, particularly where machine translation was used, although this is unlikely to affect the categorization for coding purposes here (as of 2019, Google Translate has been found to be comparable to human translation for extracting data from articles for systematic reviews, a similar goal to ours in this study [32]).

## Results

### Prevalence of actions, indicators, and outputs in urban biodiversity plans

**Fig 1:**
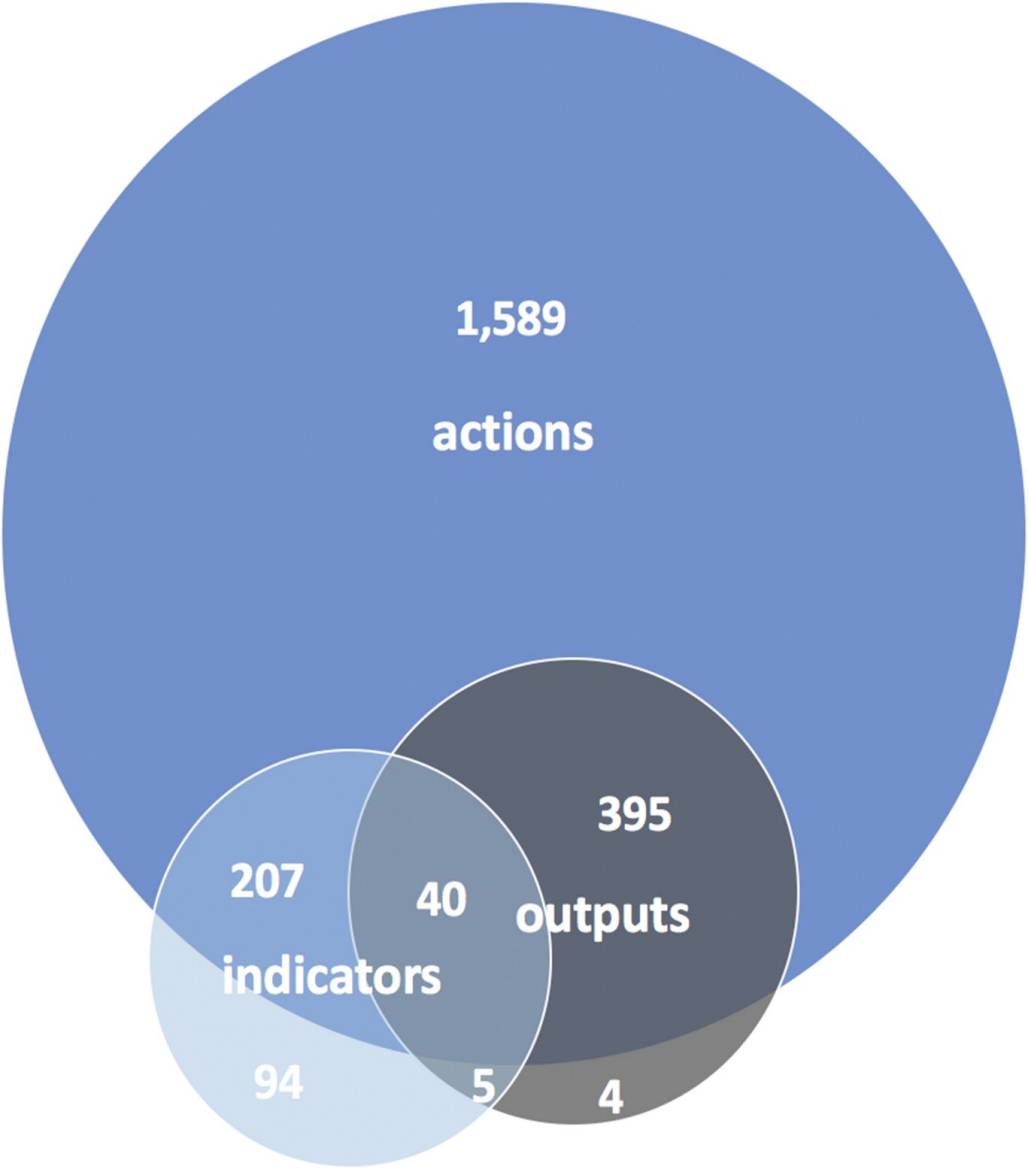
Venn diagram showing associations between actions, indicators, and outputs in biodiversity plans. Most actions lacked any measure, whereas less than one-third of indicators lacked a matching action, and nearly all outputs had matching actions. It was also rare for an action to have both types of measures. Note that overlapping areas are not proportional.

While most actions were not matched directly with measures in the plans, most measures were matched with actions. In total, we identified 3,021 plan elements, which consisted of 2,231 actions, 346 indicators, and 444 outputs across all 39 biodiversity plans (Fig 1). Of the actions, a minority (29%) had at least one directly matching measure. It was rare (2%) for an action to be linked to both an indicator and an output. Conversely, most of the measures (71% of indicators and 98% of outputs) had a matching action.

**Fig 2:**
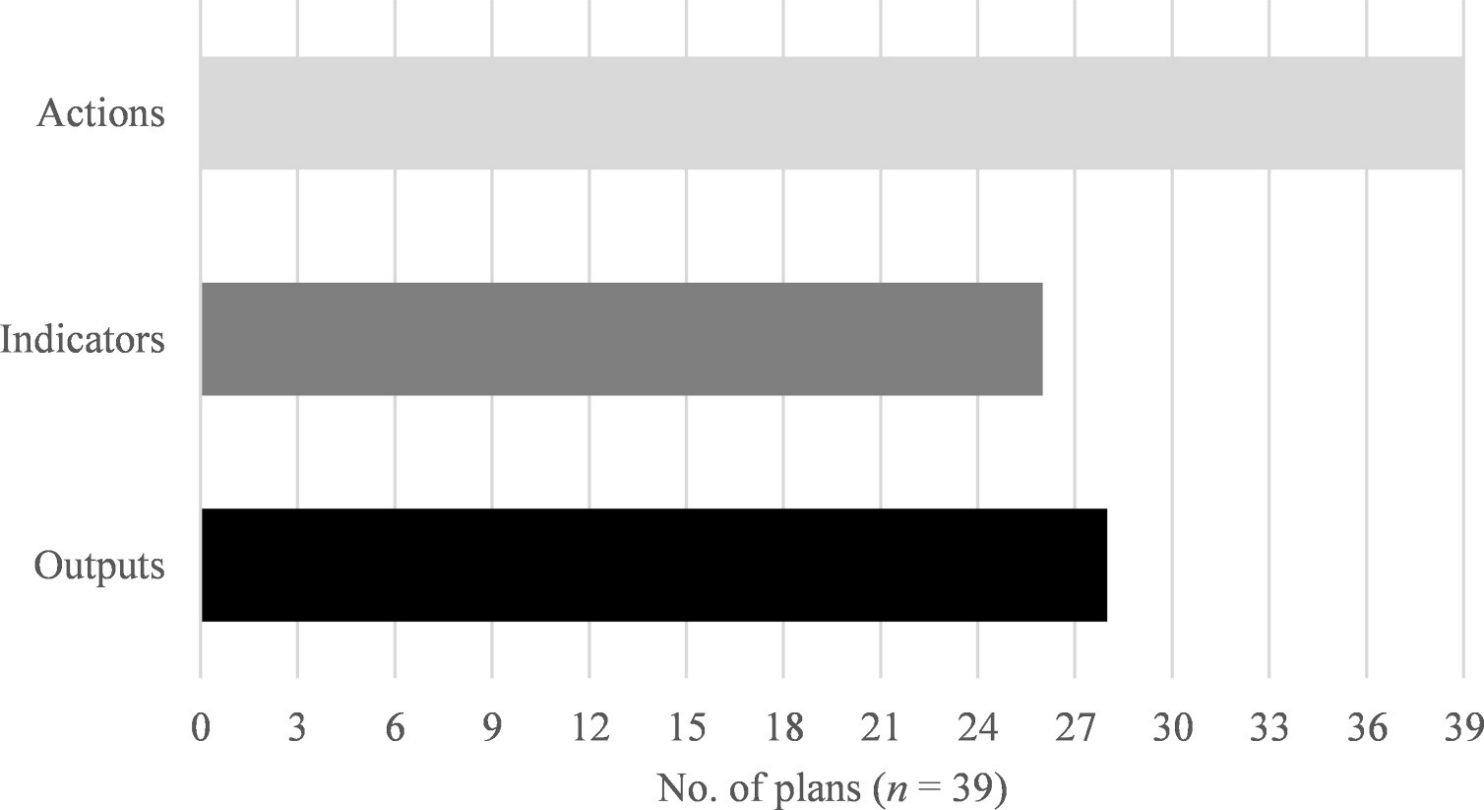
Counts of biodiversity plans with actions, indicators, and outputs. Of the 39 plans evaluated, all contained actions, 26 (67%) contained indicators, and 28 (72%) contained outputs.

We found that in their biodiversity plans, cities explicitly stated their intended actions and typically also included some type of measure for those actions, although the numbers of measures per plan varied widely (see S3 Table in the supporting information). The count of actions ranged from a minimum of five in Jerusalem to a maximum of 243 in Edinburgh. The majority of the plans (32/39, or 82%) included some intended measure for at least some actions, either in terms of a quantitative indicator (26/39, or 67% of plans) or an output (28/39, or 72% of plans) (Fig 2). The inclusion of measures was particularly uncommon in the Americas, where only 2 out of 9 cities (Calgary and Montreal) had indicators and only 5 cities had outputs.

When comparing the mean and median numbers of actions, indicators, and outputs, collectively referred to as the plan elements, we found the median to be lower than the mean for all three elements. A subset of plans containing a much larger quantity of plan elements, particularly indicators and outputs, strongly influenced the means. The biodiversity plans in the total sample (*n* = 39) had a median number of actions of 38 (mean = 57.2; range = 5 to 243). Among the plans that included indicators (*n* = 26), the median number of indicators of 8.5 (mean = 85.8; range = 1 to 49). Among the plans that included outputs (*n* = 28), the median number of outputs was 6 (mean = 79.7, range = 1 to 88). In sum, these data showed that while cities’ urban biodiversity plans did explicitly state their intended actions, explicit measures to track progress made on those same actions were less commonly included.

### Classifying actions and measures by SI indicator

The SI indicator categories effectively captured the majority of all plan elements, both overall and on average by plan. Overall, 1,454 (65%) of the actions, 260 (75%) of the indicators, and 316 (71%) of the outputs were classifiable by SI indicator category. When we adjusted for the different numbers of elements in each plan by averaging the percentages in each plan, the SI indicator categories covered 85% of actions, 98% of indicators, and 98% of outputs. Amsterdam’s plan was unique in that every element was classifiable by SI indicators 1 or 2. All other plans were broader in scope, containing elements covered by additional SI indicator categories and also having at least some elements not captured by the SI framework (listed in the “Other topics covered in urban biodiversity plans” section). When all elements in the urban biodiversity plans were coded by SI indicator, we found that each of the 18 SI indicator categories could be used to code examples of each plan element (actions, indicators, and outputs). However, each plan covered only a subset of the SI indicators. In each plan, on average, actions had the broadest coverage, followed by indicators and then by outputs.

The average urban biodiversity plan contained actions that could be classified by half of the SI indicator categories. In some biodiversity plans, the actions were narrowly focused; for example, all 133 actions in Amsterdam’s plan were classified by SI indicators 1 and 2. Other cities took a more comprehensive approach, including São Paulo, where plan actions were classified by 15/18 SI indicator categories and no more than 7 of 80 plan actions could be classified by any individual SI indicator. Edinburgh had the greatest variety of actions, with its 243 actions classified by 16 of the SI indicators.

Indicators were generally more narrowly focused than actions. Among the 26 biodiversity plans that contained indicators, we found that, on average, indicators could be classified into 4/18 (22.2%) of the SI indicator categories. The indicators within urban biodiversity plans were also varied in what they covered. Some examples of indicators include the count of the number of education projects for environmental education programming at zoos (Yokohama), the percentage of semi-natural and naturalized spaces (Lisbon), the number of sites selected as areas with rich biodiversity for possible conservation programs (Lilongwe), the number of public and private organizations that participate in biodiversity conservation activities (Singapore), and the percentage of open space restored to support the conservation of biodiversity (Calgary). However, out of the 39 urban biodiversity plans analyzed here, the Lisbon and Yokohama plans covered the greatest variety of topics through their indicators, that could be classified by 14/18 (78%) and 13/18 (72%) of the SI indicators, respectively.

As with indicators, outputs were also narrowly focused. Among the 28 plans containing outputs, we found that they could be classified, on average, into 3/18 (16.6%) of the SI indicators. Some examples of outputs in the urban biodiversity plans include the pledge to become a more ecologically connected city by 2027 than in 2017 (Melbourne), the development of a computerized database for wetlands to produce public reports on wetland targets and trends (Johannesburg), the involvement of community and land managers in habitat management and creation (Glasgow), the creation of biodiversity maps (Sapporo), the creation of a self-guided tour with georeferenced downloadable podcasts about the habitats and wildlife found in the ravines and/or along a street or transit routes (Toronto), and the creation of public seed libraries (Paris). The Paris and Zurich plans had the greatest variety of outputs, which could be classified by 11 (61%) and 10 (56%) of the 18 SI indicator categories, respectively.

**Fig 3:**
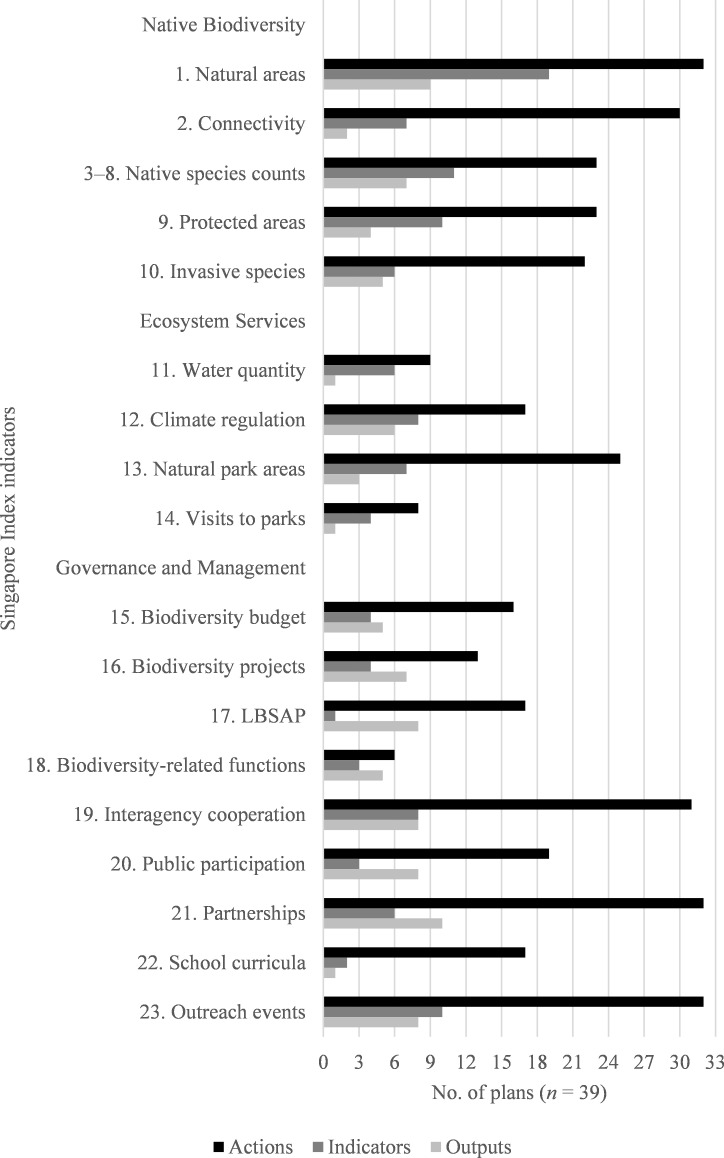
Plan elements by Singapore Index indicator. This chart compares the classification of plan elements (actions, indicators or outputs) from all 39 sampled cities according to the 23 SI indicators (indicators 3 to 8 were treated together for classification purposes) under the core components of native biodiversity (NB), ecosystem services (ES), and governance and management (GM). Note that while the majority of the plans included some actions related to most of the SI indicators, far fewer plans attempted to measure progress or outcomes using indicators or outputs.

Fig 3 shows the occurrence of plan elements (i.e., actions, indicators, and outputs) for each of the 18 SI indicator categories. For each SI indicator, the occurrence of actions in urban biodiversity plans far exceeds the occurrence of indicators and outputs in plans.

### Topics covered in urban biodiversity plans and how they are measured

In addition to displaying the occurrence of plan elements, Fig 3 also reveals the most and least frequently mentioned topics (of those classified by the SI framework) within the plans. Actions associated with the topics of natural areas (SI indicator 1), outreach (SI indicator 23), and partnerships (SI indicator 21) were among the most frequent, appearing in the majority of the sampled plans (32/29, or 82%). Meanwhile, actions associated with visits to natural areas (SI indicator 14), functions (SI indicator 18), and water quality (SI indicator 11) were among the least frequent topics covered, appearing only in 8, 6, and 9 plans, respectively. Indicators were most commonly categorized as relating to natural areas (SI indicator 1), with 19/39 (49%) of the plans having at least one natural area indicator, which is nearly double the frequency of the next most common SI indicator (3 to 8, native species counts). Outputs were also commonly classified by the SI natural areas indicator (9/39, or 23% of plans), but were most frequently classified by the SI partnerships indicator (10/39, or 26% of plans).

The relative ratios of elements within each plan by SI indicator are summarized in Table 3. Like that shown in Fig 3, this analysis revealed a high frequency of indicators measuring natural areas (SI indicator 1) in the plans and a low frequency of plan elements classified by SI indicators 11, 14, 15, 18, and 22. Within the 26 plans that contained indicators (classifiable by the SI framework and/or by additional topics), 72% of indicators overall could be classified by the SI framework, with a range of 0% (in 2 plans) to 100% (in 7 plans). Within the 28 plans that contained outputs, 55% could be classified according to an SI indicator, with a range of 0% (in 4 plans) to 100% (in 5 plans).

### Native biodiversity

The native biodiversity core component of the SI consists of SI indicators 1 to 10, which include measures of natural areas, their status and connectivity, and species presence (both native and invasive). Native biodiversity was a major focus of the plans analyzed here. All plans contained actions in this core component, 22 of the plans contained indicators, and 16 contained outputs. The most common topic we identified in city plans was natural areas (SI indicator 1). This topic was connected to at least one action in 32/39 (82%) of plans and to indicators in 19/39 plans (49%). In outputs, this topic was included in 9/39 plans (23%) (Fig 3).

The plan elements classified by natural areas (SI indicator 1) ranged from general in focus, such as the “quantity of biodiversity spaces created” (Paris), to more specific, such as “reedland coverage on banks” (Berlin). Some plans focused on outcomes to rehabilitation efforts, such as “degraded sites along riverbanks rehabilitated” (Lilongwe), while others covered maintenance of existing areas, such as “urban meadow sites maintained” (Glasgow). Some also included related issues such as connectivity or access, e.g., “number of locations of green implementation in areas that lack green areas” (Yokohama).

**Fig 4:**
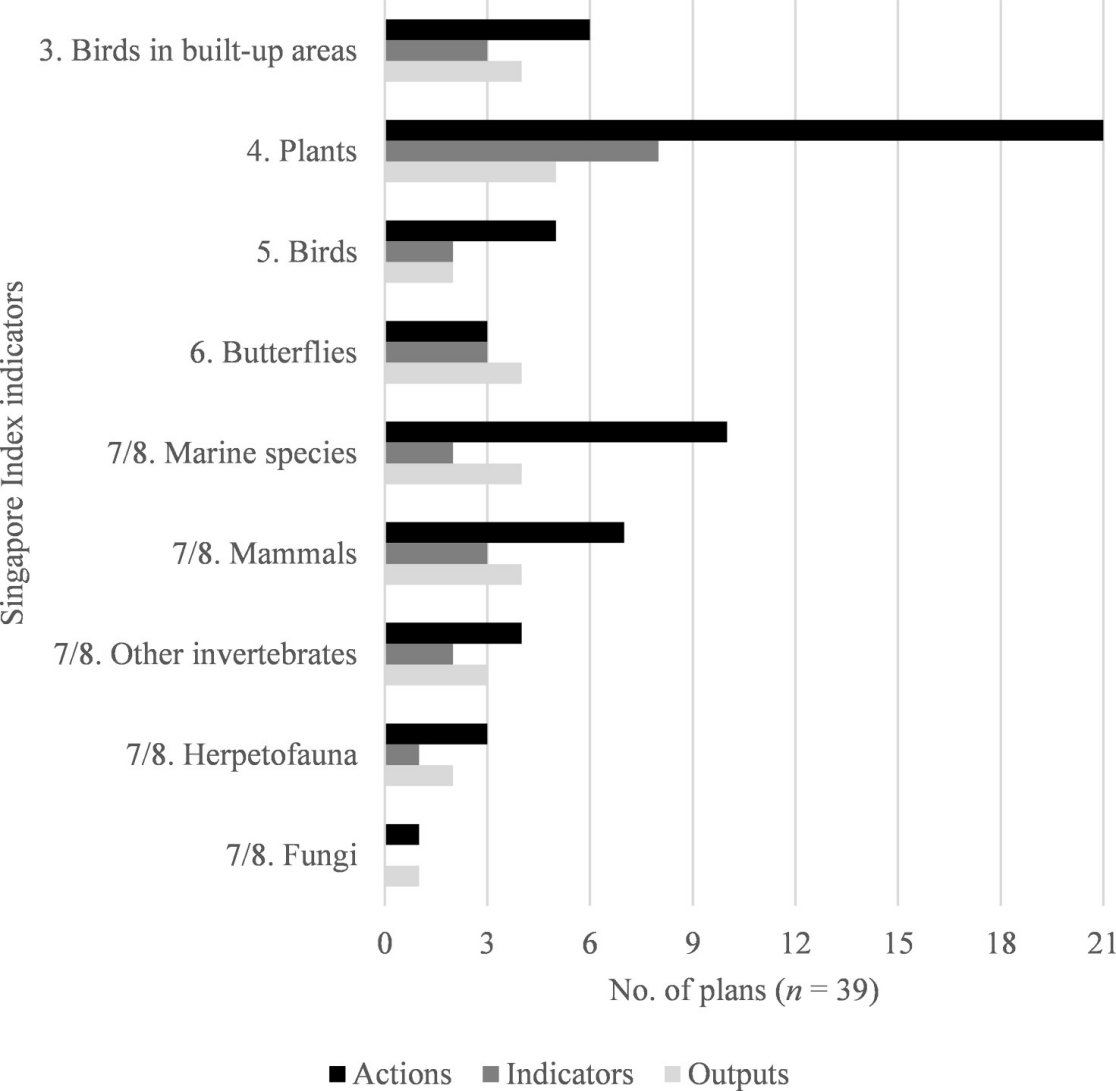
Taxonomic categories measured in urban biodiversity plans. The Singapore Index includes indicators specific to birds in built-up areas, plants, birds, and butterflies (3 to 6), then leaves the taxonomic group for indicators 7 and 8 open for cities to select [21]. Indicators for five specifically named taxonomic or ecological groups (marine organisms, mammals, herpetofauna, fungi, and invertebrates other than butterflies), which we categorized by SI indicators 7 and 8, were also found in the city plans. Note that we also categorized plan elements concerning artificial nest sites and bird baths by SI indicator 3, birds in built-up areas.

The next most common topic in the NB component covered by plans was native species counts (SI indicators 3 to 8). Across all plans, there were 172 actions, 41 indicators, and 17 outputs classified by SI indicators 3 to 8 (Fig 4).

In selecting organismal groups to measure or monitor, city plans most often focused on plant species. This focus was consistent for all three types of plan element, with 21 plans containing actions about plants, 8 of them containing indicators, and 5 containing outputs. Marine species were the next most common group discussed, followed by a tie between birds and mammals. Butterflies and other invertebrates were among the least commonly mentioned in plans. Fig 4 shows a breakdown by original SI indicator classification of specific taxonomic categories measured in the urban biodiversity plans.

In the native biodiversity core component, the second most common classification for actions was SI indicator 2, connectivity. Connectivity is the degree to which the landscape facilitates or impedes movement among habitats or resource patches [33], but in the urban context, in particular, habitat fragmentation and the presence of physical features (e.g., tall buildings) that impede species movement are of particular concern for urban wildlife [34]. This topic, although it was included in many urban biodiversity plans, was less frequently measured quantitatively, with only 7 indicators across plans, and was more frequently measured qualitatively, with 93 outputs across plans (Fig 3).

Measures regarding protected natural areas were typically concerned with achieving protected designation status or with the management of significant areas. They were measured by number of areas or by land area under protection.

Indicators and actions concerning invasive species tended to focus on the measurement of range, population, and/or the quantity removed/captured of a particular species. These varied widely, ranging from regulation of trade of exotic invasive plant species, as in São Paulo, to monitoring the status of invasive species in general, as in Hong Kong, to particular actions focusing on a single invasive species, as in Cape Town. Outputs included publication of management plans or maps or the adoption of management strategies.

### Ecosystem services

The ecosystem services core component of the SI consists of SI indicators 11 to 14, which include measures related to stormwater infiltration and management, climate regulation in the form of addressing the urban heat island effect and carbon storage, and recreational opportunities that are afforded by biodiversity in urban areas. Cities demonstrated interest in a wider range of ecosystem services than those covered by the SI; these other ecosystem services are discussed below in “Other topics covered in urban biodiversity plans.”

SI indicator 11, water quantity, is calculated based on the percentage of land area that is both permeable and not “artificial” [21]. Four out of the six water quantity indicators found in city plans measured stormwater management by the amount of permeable area. However, measures based on this concept defined permeability in various ways (i.e., by limiting the measure to vegetated areas or to public spaces).

SI indicator 12 combines the impact of vegetation on both carbon sequestration and cooling. Carbon sequestration was most often measured by tree planting, but there were also several plans that took into account other measures, including carbon sequestration in wetlands. The urban heat island effect was addressed by a variety of greening methods, including green roofs and walls, as well as by street trees (note that green infrastructure plan elements with other or unspecified purposes are discussed below in “Other topics covered in urban biodiversity plans”).

Natural park areas were typically measured as a percentage of parks, total area, or the number of park naturalization projects completed. Outputs included the opening of biodiversity-oriented areas or the creation of guides or maps indicating such areas to the public. Visits to parks were measured as the number of visitors overall or of children in particular; by user satisfaction with parks, educational awards; and by the creation of outdoor activities or biodiversity spaces on school grounds.

### Governance and management

The governance and management core component of the SI consists of SI indicators 15 to 23, which include measures related to financial resources and projects devoted to biodiversity, as well as efforts to integrate biodiversity across city agencies and departments and opportunities to engage the public and institutions to help raise awareness.

Various approaches were taken by each city to measure the governance and management of biodiversity. We identified no particular patterns in these approaches. Measures in biodiversity plans classified by SI indicators 15 to 23 are summarized in more detail in Table 4; for a complete list of these plan elements, see raw data in S1 Dataset in the supporting information.

**Table 4:** Measures for governance and management.

SI indicator	Measures found in biodiversity plans
**15. Budget allocation for biodiversity**	Funding indicators included budgetary commitments, staffing strategies, the number of jointly funded initiatives, the number of funded projects, and funds obtained from outside sources.
**16. Biodiversity projects**	Measured by the number of projects implemented, which may be undefined or be of a particular type, ranging from beehive programs to partnership projects to new Web databases.
**17. LBSAP**^**a**^	Most of these measures were outputs that would support a Local Biodiversity Strategy and Action Plan (LBSAP), such as a conservation plan for a particular area or species or a conservation communications plan. One city’s plan also called for measurement of areas restored as a result of a green and blue plan.
**18. Biodiversity-related functions**	Measures included the number of lectures and exhibitions, as well as establishment of biodiversity-oriented exhibits, civic institutions, grain banks, or training centers.
**19. Interagency cooperation**	Many ways to measure interagency cooperation were identified, but the most common was a count of cross-departmental or cross-council initiatives. Some were also linked with funding, integration of biodiversity initiatives within plans by other departments or at higher government levels, or participation in commitments or reporting at a larger scale. Some measured the ongoing status of existing programs.
**20. Public participation**	There was stated interest in this topic but not much effort to measure participation. Those plans that called for measurement of actions in this topic area did so by counting the number of community groups or area of land under community management. Others counted volunteer programs or the establishment of data-sharing platforms with volunteers.
**21. Partnerships**	A variety of measures ranged from the creation of partner networks and co-published resources to the frequency of events or meetings with particular groups. Some plans called for measuring this more directly through a percentage or count of partnering institutions.
**22. School curricula**	Two plans included indicators; one city counted the number of schools with a particular educational program, and the other counted the number of schools with a naturalized outdoor area. In terms of outputs, one city intended the establishment of a comprehensive biodiversity curriculum, activities, or resources across city schools.
**23. Outreach events**	Outreach events were primarily expected to take place at zoos, parks, schools, or similar institutions. Some events were part of awareness campaigns, while others were more education focused. Measures generally focused on event frequency or on counting the number of event sites, but could also be measured by the number of institutions hosting events.

^a^LBSAP, Local Biodiversity Strategy and Action Plan.

### Other topics covered in urban biodiversity plans

The 39 biodiversity plans in this study also included topics besides those that could be classified by the SI framework as discussed above. In total, we inductively derived a total of 20 additional categories into which we grouped these plan elements. Not surprisingly, these other categories were also varied and included planning/management, socioeconomic considerations, data collection outside the scope of the SI framework, genetic diversity, pollution, production/consumption, wildlife conflicts (i.e., conflicts between wildlife and humans or their pets), urban agriculture, traditional culture and indigenous knowledge, and citizen science, among others.

While these other topic areas differed from the ones already discussed above in this paper, they shares the same general pattern in that the total count of actions (*n* = 777) far exceeded the number of measures used to track progress in the form of outputs (*n* = 128) and indicators (*n* = 86).

**Fig 5:**
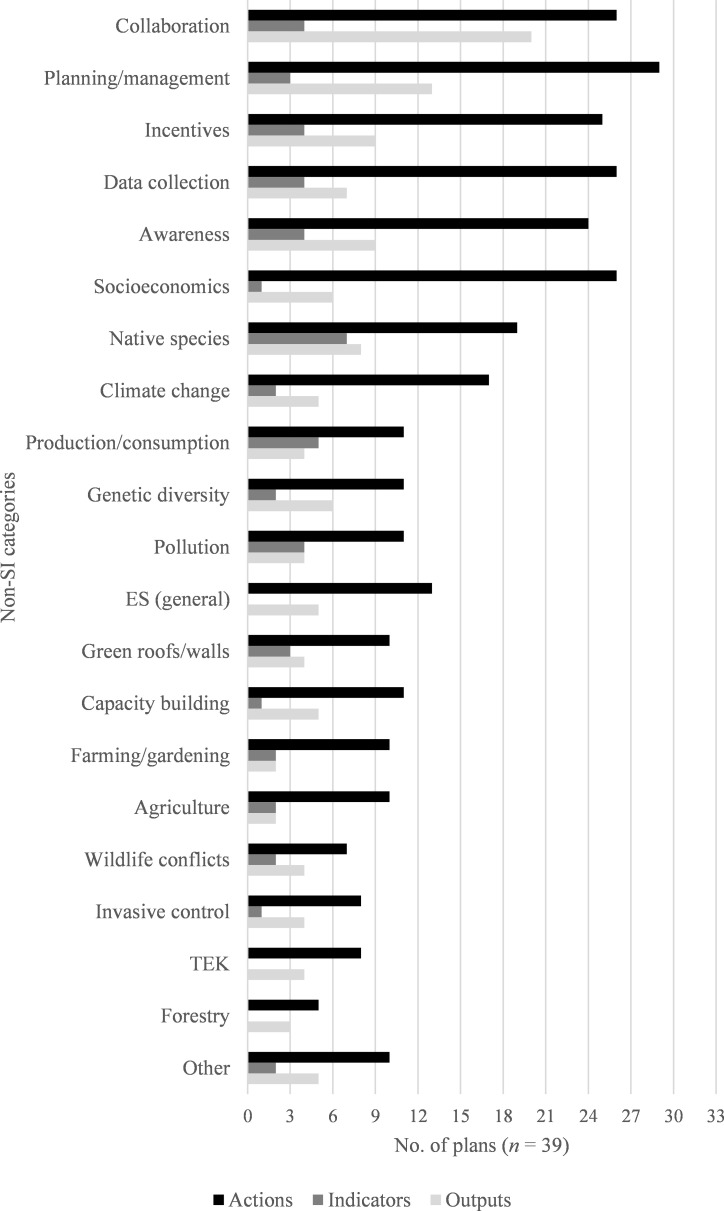
Plan elements classified by inductively derived topic. Chart of the categories for all plan elements that were classified according to inductively derived topics once it was determined that they could not be classified according to the SI framework. “Native species” here refers to plan elements related to native species other than species count indicators. ES, ecosystem services; TEK, traditional ecological knowledge.

Fig 5 shows the distribution of plan elements across the 39 biodiversity plans among the 20 additional topic areas. In more than half of the plans, most actions related to these additional topic areas were classified as planning/management (29 plans), collaboration (26 plans), socioeconomic (26 plans), data collection (26 plans), incentives (25 plans), and awareness (24 plans). The most frequently measured topic was collaboration initiatives (20 plans); for other topic areas, only a few plans per topic included outputs.

Indicators were less frequently used to measure actions associated with these categories than to measure actions classified by the SI. The topic area covered most frequently by indicators was native species, with 7 biodiversity plans including indicators for native species in general (differentiated from indicators in plans that could be classified by SI indicators 3 to 8, which each refer to a particular organismal group rather than to species assessment and monitoring in general). Meanwhile, no indicators were used to measure progress for the forestry and general ecosystem services topic areas. Even though outputs were more frequently used than indicators in urban biodiversity plans to measure actions in topic areas not covered by the SI, both types of measure were only used in a very limited way for gauging progress in these areas.

Plan elements that could not be classified by SI indicator were often very specific to local context and/or concerned topics of public interest, such as combining educational opportunities and the harvest of material resources through urban forestry and agriculture. In other cases, cities expressed interest in supporting a wider variety of ecosystem services than those covered by the SI framework (also noted previously by Kohsaka et al. [ 26]).

Another key topic that could not always be classified by SI indicator was green infrastructure, such as green roofs and walls. In some cases, plan elements related to this topic could be classified as climate regulation (SI indicator 12) or as related to governance and management indicators (e.g., interagency cooperation [SI indicator 19] or partnerships [SI indicator 21]), but in most cases, actions related to green infrastructure referred to general greening of public and/or private spaces. With one exception (São Paulo), cities that explicitly mentioned green infrastructure in their actions were located in Europe or Canada; one European city (Barcelona), framed its plan as a green infrastructure plan.

## Discussion

### Lack of measurable actions in urban biodiversity plans

Action statements were a standard and consistent component of all the biodiversity plans, but measures were both less common and less consistently linked to actions. The paucity of indicators and outputs in relation to the number of actions in urban biodiversity plans as shown in Fig 1 and in S3 Table in the supporting information is consistent with the findings of Nilon et al. [18] and Stossel et al. [19] who similarly noted the lack of accountability measures in biodiversity plans. This lack may be due in part to political challenges. Actions are statements of what the government is going to do and are a core component of plans and strategies designed to address a known problem. Measures, on the other hand, may be perceived as an optional component of plans. Furthermore, while actions can be expressed in very general terms, measures call for more specificity and technical input, which means they can be more challenging to commit to or achieve consensus on among decision-makers. Accountability measures may also carry the risk of highlighting failure when local governments do not or cannot undertake the stated action(s) for biodiversity. Conversely, measures can showcase success when cities achieve the actions to which they committed for biodiversity. Thus, a lack of measures can result in lost opportunity to highlight biodiversity successes.

Among measures, indicators were less common and tended to be less directly linked to actions than outputs. The lack of indicators in particular may also be due to the lack of access to technical knowledge and experience in developing measures that are specific to urban biodiversity. Although slightly more frequently included than indicators, outputs were often embedded within actions, suggesting that city planners may not generally think of outputs and actions as separable, whereas the higher proportion of independent indicators may imply that indicators are more difficult to link directly with actions in practice. Regardless of the underlying reasons, the lack of consistent links between measures and actions in urban biodiversity plans is problematic because without a linked measure for stated actions there are no objective means of assessing the completion, success, or status of that action that would contribute to government transparency and accountability and provide technical feedback essential to improving biodiversity planning outcomes.

While our study did not investigate the reasons for the differences in frequency of types of plan elements, the gap between actions and measures suggests that is easier to express intended actions for biodiversity than it is to select measures for those actions and that outputs are often easier to define than indicators.

### Singapore Index: A useful but incomplete framework

Our research suggests that while the SI can serve as a relevant framework for urban biodiversity planning, it does not provide a complete description of relevant elements. The SI framework covered the majority of the plans overall and, in particular, described indicators well. However, gaps in the SI were also confirmed by the fact that the majority of the plans also list additional actions, indicators, or outputs not covered by the SI. Some of the gaps were due to indicators that had been too narrowly defined. For example, plans addressed “climate change” in more varied ways than SI indicator 12 (climate regulation), which focuses solely on carbon sequestration through urban vegetation. Other gaps were due to topics that were included in the plans but are not included in the SI. We therefore suggest supplementing the SI-based framework for future research along these lines, and would also suggest some expansion of the SI indicators in scope, if not in number, for future iterations of the SI.

### Native biodiversity is the predominant focus

As the most obviously connected to biodiversity and the most traditional approach to biodiversity conservation, it is no surprise that actions and measures related to native biodiversity were the most common and consistent component of biodiversity plans.

Within the native biodiversity core component, “natural areas” was the most common topic. The SI defines “natural areas” by a predominance of native species and low human influence but does not differentiate natural habitat from restored or reconstructed habitat [21]. Unlike the SI indicator, indicators in plans tended to differentiate between restored areas and preexisting natural sites to be maintained. They also frequently referred to specific ecosystems rather than grouping all “natural areas” together. This may reflect the utility on the local level of greater ecological specificity, as well as the fact that not all cities have remaining “natural” areas by the SI definition and thus may find other categories more useful in practice [26].

The frequent inclusion of natural areas in the plans may be due to the fact that green space and park management is a standard component of city governance. Increasing the size or number of green spaces may have already been a pre-existing part of city plans, requiring little adjustment to be included in biodiversity plans. It is relatively straightforward in principle to increase the size and quality of natural areas in the city (e.g., by planting more native trees). It is also a standard part of city data gathering to measure the extent of such areas, especially with advancements in and increased access to remote sensing technology. Other topics within this core component, such as increasing the connectivity of natural habitats; conducting species counts or otherwise determining species abundance, richness, and density; and the control of invasive species, are generally more complicated and expensive to implement and measure. This is reflected in their lower frequency of coverage in the plans by all elements (actions, indicators, and outputs) when compared with natural areas.

Ecological connectivity was the second most common topic in this core component when it came to actions, but it was nearly in last place when it came to measures. Although the high frequency of actions indicate there is interest in ecological connectivity, this lack of measurement strategies may reflect the relative difficulty of defining and measuring ecological connectivity [35, 36] compared to area- and species count-based measurements. A standard connectivity indicator could be an important part of a more complete index for urban biodiversity.

Native species-related actions and measures generally focused on counting the frequency of species, either overall or within a certain category (i.e., plants, marine taxa, or birds). The groups of native species selected varied by city. Birds and plants were among the most commonly mentioned, likely due to the relative ease of identifying and counting them as compared with invertebrates. However, even when selecting taxa that are “easier” to measure, data reliability and availability are a challenge. Reliance on citizen science can complicate data accuracy concerns, though it must be noted that volunteer-based data gathering can serve additional purposes, such as awareness raising [37] and education. Overreliance on simple indicators like species counts to measure biodiversity can distort results because they may not be the most accurate measure of biodiversity [38, 39], because species lists tend to be cumulative, making it difficult to determine changes over time [26], and because it can be complicated to link impacts with conservation activities [40], particularly in urban environments, which can have many rapidly changing variables [41].

### Linking ecosystem services and biodiversity goals

Ecosystem services, defined as the benefits to humans from ecosystems [42], have attracted increasing attention in urban contexts, most recently for their role in nature-based solutions (e.g., Nesshöver et al. [8]). Despite the linkages between biodiversity and ecosystem services drawn by the Intergovernmental Panel on Biodiversity and Ecosystem Services and by academic researchers (e.g., see the volume edited by Elmqvist et al. [43]), of the three SI core components, ecosystem services were the least commonly incorporated into the plans. This may be in part due to difficulty in connecting the two concepts in an easily understandable way. This difficulty increases when it comes to finding ways of measuring ecosystem services while maintaining a meaningful link to biodiversity [44]. For example, although 27% of the urban biodiversity plans included actions pertaining to water quantity and only 18% included indicators (Fig 3), the role of biodiversity in the management of stormwater (or water quantity, as in the SI) is widely acknowledged [45–48]. However, the method of measurement can be an important factor. Managing stormwater was often measured by amount of permeable area, which can be achieved with or without contributing to biodiversity directly, and which, depending on the groundwater conditions, may or may not indirectly contribute to ecosystems through greater access to water. None of the plans differentiated areas where permeability might be most beneficial to people (such as in flood-prone areas) or to ecosystems (such as areas prone to saltwater intrusion) in their measures. Similar criticisms could also be made of the need to more clearly link other ecosystem services to biodiversity benefits when referenced in biodiversity plans.

The four SI indicators in this core component account for the following ecosystem services: stormwater quantity management (SI indicator 11), climate regulation (SI indicator 12), and access to nature (SI indicators 13 and 14). These four SI indicators capture just a selection of the full array of ecosystem services to which biodiversity can contribute in cities [26]. Some city plans cover these ecosystem services or even a wider array than the SI indicators, including ecosystem services related to water quality, food production, pollination, and carbon sequestration. While many of the ecosystem services actions and measures in the plans are linked to biodiversity, they can sometimes require careful execution to maintain these linkages. For example, planting trees for cooling and thermal insulation (in the Birmingham plan) can be done so as to maximize either shade for cooling or habitat for biodiversity. Some actions, such as reducing air conditioning by using natural light, wind, or plants (in the Nagoya plan), do not have direct biodiversity benefits as goals. Thus, focusing on goals related to ecosystem services may not adequately address biodiversity loss, even though there are synergies between the two [49]. Therefore, greater specificity would be beneficial when attempting to integrate potentially unrelated goals into a biodiversity plan to clarify how decisions are to be made and to differentiate a biodiversity plan from other types of plans, such as climate action plans. Further research is needed to explore how cities are addressing these sometimes-conflicting goals to clarify how decisions are to be made and maximize synergistic opportunities. Research results also need to be translated into guidelines so that linkages between biodiversity and ecosystem services can be further incorporated into practice.

### Governance measures lack specificity and consistency

The governance and management core component of the SI is focused on socio-economic activities, which have been described by Folke [50] in Borgström [51] as an essential part of urban biodiversity planning. From committing financial resources to seeking cooperation among city departments to engaging the public on biodiversity issues, these social processes represent a varied and complex mix of commitments. For actions associated with the governance and management SI indicators, more city plans chose outputs rather than indicators as their accountability measure (Fig 3). In the realm of the political and the social, it is perhaps easier to showcase a tangible output as a measure of success, whether that is the creation of a local biodiversity plan or the establishment of a network. Therefore, any future indices may wish to remain flexible in regards to measuring actions in these categories and to consider outputs as well as indicators to inform the index.

### Emerging topic areas in urban biodiversity plans

While not constituting the majority of the actions, indicators, or outputs of the urban biodiversity plans in this study, additional topic areas outside of the SI indicator categories included in the plans, such as urban agriculture and forestry, human wildlife conflicts, wildlife trafficking, traditional culture and indigenous knowledge, socioeconomic opportunities, and green infrastructure, demonstrates that cities are incorporating concepts beyond those represented by the SI. While cities seem to have confidence in making action statements on these topics in their plans, there is less confidence in stating measures, judging by the relatively low ratio of measures to actions in topics outside of the SI indicator categories (Fig 5). This gulf between the number of actions and measures may speak to the novelty of (and therefore a lack of technical support for) these topics as they relate to urban biodiversity. This finding reinforces a similar finding by Kohsaka et al. [26], who found that measures of biodiversity in an urban context lag behind scientific knowledge of their linkages to human wellbeing due to the complexity and interdisciplinarity of socio-ecological systems. Where measures are included for the actions of these other topic areas, outputs outnumber indicators, suggesting that it is easier to showcase and highlight a deliverable (e.g., the production of an educational guide) that results from undertaking an action than it is to use a quantifiable indicator. Nevertheless, the diversity of these topics suggests that cities are attempting to address connections between urban biodiversity and diverse areas that have been suggested in previous research [49, 52, 53], even in the absence of formal indices or indicators to guide them.

## Conclusions

All 39 biodiversity plans analyzed in this study explicitly stated intended actions, and the majority (82%) also included some measure of their actions, either in the form of quantifiable indicators (67%) or outputs (72%).

Most (71%) of the plan’s action statements were not linked with accountability measures, although almost all (98%) of outputs and most (71%) of indicators were linked with actions. Therefore, cities seemed to be more ambitious when it came to action statements, but less able or willing to link some of those statement to accountability measures. Further research will be necessary to identify the reasons behind lack of measurement for urban biodiversity actions and to develop better supports and incentives for cities to measure their actions.

All 18 of the Singapore Index (SI) indicator categories were represented in the actions, indicators, and outputs in the plans. The SI framework was representative of the majority of each of these plan elements (percentages of actions, indicators, and outputs). However, all but one of the plans also contained actions and other elements that were not covered by the SI indicator categories. The actions in the plans outside of the SI indictor framework had lower ratio of measures to actions. Therefore, the SI framework is a helpful, but somewhat incomplete framework for urban biodiversity plans in practice. It is most effective as a framework for accountability measures in biodiversity plans rather than actions. The additional topics we identified in the urban biodiversity plans could inform the revision of the Singapore Index or the development of new urban biodiversity frameworks, specifically by suggesting areas where indicators may be revised or added to better reflect and capture the initiatives on which cities are embarking for biodiversity. Since one of the criticisms of the SI has been that it has “too many indicators” to be easily applied [26], consideration should also be given to merging indicators with significant overlaps in intent, such as the native species count indicators (3 to 8), although cities would have the option of using relevant subindicators as desired.

Topics covered by the biodiversity plans included all of those covered by the Singapore Index indicators, plus additional topics that emerged from the plans themselves, although some plans were narrower in scope than others. All three core components of the Singapore Index (native biodiversity, ecosystem services, and governance and management) were found in all but one plan. Native biodiversity was the most common of the SI core components in city plans. City plans went beyond the topics captured by the SI framework to include urban agriculture, management of human-wildlife conflicts, green infrastructure, data collection, traditional culture and indigenous knowledge, and socioeconomic measures.

Where used, the measures in urban biodiversity plans varied widely, and no standardized indicators shared between cities were identified. While local context is important, and dissimilar cities may not benefit particularly from using identical indicators, the development of a range of indicators that cities can choose from may facilitate measurement and monitoring over time.

As the urban population increases rapidly in the near future, and against a backdrop of the urgent need for sound management and conservation of our natural environment, cities are increasingly called to improve the quality of life for their residents and to make measurable contributions to global environment goals. The emerging implications of the COVID-19 global public health crisis for the future of urban planning and public space design, which are just entering the discussion (e.g., Honey-Rosés et al. [54]), also emphasize the importance of green spaces and biodiversity to public health and raise questions of how to best incorporate biodiversity into the urban landscape. The development of the post-2020 global biodiversity framework thus presents an unprecedented opportunity for meaningful integration of subnational and local contributions to urban biodiversity. Our work here offers a first step towards understanding how cities plan for biodiversity in practice. Additional research on the effectiveness of particular actions and metrics is needed to suggest which actions and measures could be standardized for comparability across diverse contexts and under what conditions this would be beneficial. Ultimately, these actions and measures at the local level should be coordinated with those at the global scale, so as to catalyze and monitor efforts at all levels in our collective race against time to halt biodiversity loss and secure a viable planet for future generations.

## Supporting information

S1 Dataset Coded action, indicator, and output data from the 39 plans analyzed in this study. The “City plan analysis” tab contains the text of the coded actions, indicators, and outputs extracted from the individual plans. The “code” column contains the SI indicator number or additional codes defined in the “Metadata” tab. The “code_type” column indicates whether a plan element was coded by the SI framework (“SI”) or by inductively derived categories (“n/a”) (see “Other topics covered in urban biodiversity plans” in the Results).
(XLSX)

S1 Table City metadata (name, location, and World Bank region) and titles and publication years of the 39 urban biodiversity plans analyzed in this study. (XLSX)

S2 Table Simplified structure of the Singapore Index used for coding urban biodiversity plan elements. (PDF)

S3 Table Total numbers of actions, indicators, and outputs in each urban biodiversity plan. (PDF)
