# Supplementary material for: Actions, indicators, and outputs in urban biodiversity plans: A multinational analysis of city practice
Source: PLoS One. 2020 Jul 8;15(7):e0235773. doi: 10.1371/journal.pone.0235773 (PMC7343153; doi:10.1371/journal.pone.0235773)
Supplement: S2 Table — (PDF) [file pone.0235773.s003.pdf]

S2 Table. Simplified structure of the Singapore Index used for coding urban biodiversity plan elements.

Core component[a]	Indicator no.	Indicator topic
**Native biodiversity**	1	Natural areas
	2	Connectivity
	3 to 8[b]	Native species counts
	9	Protected natural areas
	10	Invasive species
**Ecosystem services**	11	Regulation of water quantity
	12	Carbon storage and cooling effect of vegetation
	13	Natural park areas
	14	Visits to natural areas
**Governance and management**	15	Budget allocation for biodiversity
	16	Biodiversity projects
	17	Local Biodiversity Strategy and Action Plan (LBSAP)
	18	Biodiversity-related functions
	19	Interagency cooperation
	20	Formal or informal public participation
	21	Partnerships
	22	School curricula
	23	Outreach/public awareness events

[a]The Singapore Index's 23 indicators are arranged into three core components (summarized from Chan et al. [21]).

[a]Although we coded plan elements by each separate indicator, for the purposes of our analysis, we combined SI indicators 3 to 8 into a "native species counts" indicator. See "Coding protocol and analysis" in the main text for more detail and justification of this approach. We classified plan elements by SI indicator 3, which covers bird species counts in built-up areas, if they pertained to bird species in more human-centered habitats, e.g., quantification or support of artificial bird nests.
