# Supplementary material for: Actions, indicators, and outputs in urban biodiversity plans: A multinational analysis of city practice
Source: PLoS One. 2020 Jul 8;15(7):e0235773. doi: 10.1371/journal.pone.0235773 (PMC7343153; doi:10.1371/journal.pone.0235773)
Supplement: S3 Table — (PDF) [file pone.0235773.s004.pdf]

S3 Table. Total numbers of actions, indicators, and outputs in each urban biodiversity plan.

City	Country	No. of		
		Actions	Indicators	Outputs
**Amsterdam**	Netherlands	133	0	88
**Auckland**	New Zealand	68	49	1
**Barcelona**	Spain	66	9	16
**Berlin**	Germany	37	1	0
**Birmingham**	England, UK	21	0	0
**Bogota**	Colombia	25	0	0
**Calgary**	Canada	22	1	1
**Cape Town**	South Africa	138	6	6
**Chicago**	USA	13	0	1
**Christchurch**	New Zealand	85	15	38
**Copenhagen**	Denmark	28	8	8
**Dublin**	Ireland	54	1	6
**Edinburgh**	Scotland, UK	243	15	11
**eThekwini (Durban)**	South Africa	131	1	71
**Glasgow**	Scotland, UK	97	13	14
**Hamburg**	Germany	27	5	0
**Hong Kong**	China	67	11	5
**Jerusalem**	Israel	5	0	0
**Johannesburg**	South Africa	45	0	2
**Leeds**	England, UK	28	8	0

City	Country	No. of		
		Actions	Indicators	Outputs
**Lilongwe**	Malawi	38	30	19
**Lisbon**	Portugal	38	21	2
**London**	England, UK	71	19	6
**Medellin**	Colombia	26	0	6
**Melbourne**	Australia	23	6	6
**Mexico City**	Mexico	27	0	0
**Montreal**	Canada	28	6	0
**Nagoya**	Japan	83	0	0
**Oslo**	Norway	12	0	0
**Paris**	France	128	42	59
**San Diego**	USA	33	0	0
**São Paulo**	Brazil	80	0	5
**Sapporo**	Japan	35	8	2
**Shanghai**	China	17	1	1
**Singapore**	Singapore	25	14	5
**Sydney**	Australia	44	7	4
**Toronto**	Canada	23	0	15
**Yokohama**	Japan	72	26	13
**Zürich**	Switzerland	95	23	33
**Total for all cities**	N/A	2,231	346	444
